# Psychometric evaluation of the e-cigarette knowledge, attitude, and practice questionnaire among young students: a structural equation modeling approach

**DOI:** 10.3389/fpubh.2026.1774793

**Published:** 2026-02-12

**Authors:** Yating Wen, Jie Chen, Heng Bai, Ronald Hartono, Chaofang Yan, Zaixue Mu, Ying Chen, Rui Deng

**Affiliations:** 1 School of Public Health, Kunming Medical University & Yunnan Provincial Key Laboratory of Public Health and Biosafety, Kunming, Yunnan, China; 2 Yunnan Key Laboratory of Cross-Border Infectious Disease Prevention and New Drug Development, Kunming, Yunnan, China; 3 Xiangya School of Medicine, Central South University, Changsha, Hunan, China

**Keywords:** e-cigarette, psychometric evaluation, questionnaire development, structural equation modeling, young students

## Abstract

**Background:**

The growing popularity of e-cigarettes among young students has emerged as a pressing public health concern. Misconceptions about e-cigarettes persist widely, yet standardized tools to systematically evaluate youth knowledge, attitudes, and behaviors are limited. This study aimed to develop and validate a questionnaire evaluating e-cigarette knowledge, attitude, and practice among young students using a structural equation modeling framework.

**Methods:**

An initial e-cigarette knowledge, attitude, and practice questionnaire was developed and refined using classical test theory, including item analysis and exploratory factor analysis. A sample of 1,400 students undergraduate students were recruited from Kunming, China and Jakarta, Indonesia, yielding 1,333 valid responses (Kunming, *n* = 631; Jakarta, *n* = 702). Reliability was assessed using Cronbach’s *α*, and model fit was examined through confirmatory factor analysis within the SEM framework.

**Results:**

The final questionnaire retained 15 items: knowledge (4), attitude (8), and practice (3). Pearson correlation coefficients between each item and its respective dimension exceeded 0.4. Except for items P2 and P3, *t*-test results between high- and low- scoring groups were statistically significant (*p* < 0.05). The final model (M3) demonstrated excellent fit indices (*χ^2^/df* = 1.350, *RMSEA* = 0.050 [*90% CI*: 0.022–0.072], *SRMR* = 0.0643, *CFI* = 0.968, *TLI* = 0.960), indicating optimal model parsimony and goodness of fit. The overall Cronbach’s *α* was 0.643, with subscale coefficients of 0.819 (knowledge), 0.847 (attitude), and 0.775 (practice). Exploratory factor analysis confirmed the expected three-factor structure, with factor loadings between 0.521 and 0.819 and a cumulative variance explanation of 61.943%.

**Conclusion:**

The final instrument demonstrated acceptable reliability and validity within the sampled populations from China and Indonesia. This tool provides a practical and evidence-based approach to assess young students’ perceptions and attitudes towards e-cigarettes in public health research.

## 1 Introduction

E-cigarettes, first developed by Chinese engineer Han Li, are electronic devices designed to simulate traditional smoking behavior. These devices heat a liquid solution containing nicotine and flavoring agents, producing an aerosol that can be inhaled (1). Due to their sleek design, diverse flavors, and marketing claims as a “reduced-harm” alternative to conventional cigarettes, e-cigarettes have rapidly gained popularity worldwide, particularly among young people (2). Since 2014, e-cigarette use has evolved into a significant global public health concern, with increasing prevalence among young students (3). In North America, for instance, approximately 11.5% of adults reported smoking cigarettes and 4.5% reported using e-cigarettes in 2021 (4). Among youth, use rates are higher: 27.5% of U.S. high school students and 10.5% of middle school students reported e-cigarette use in 2019 (5, 6), declining slightly to 19.6% in 2020 (7). In the United Kingdom, 8.9% of young students used e-cigarettes in 2018 (8), while the prevalence across Europe was lower than in North America (9). In China, 2.7% of junior high school students and 3.0% of senior high school students reported e-cigarette use by 2019 (10).

Despite the increasing prevalence of e-cigarette use, public understanding remains limited and is often influenced by marketing narratives emphasizing potential benefits such as smoking cessation, absence of tar, and lower toxicity than conventional cigarettes (11). Social media further amplifies these misperceptions by portraying e-cigarettes as safer and less addictive, with minimal discussion of potential harms (12, 13). However, a growing body of evidence points to multiple associated health risks, including nicotine poisoning, device-related burns and explosions, and adverse effects on the gastrointestinal, cardiovascular, cerebrovascular, and nervous systems (14–16). The heating elements of e-cigarettes, often composed of nickel-chromium alloys, release metal particles into the aerosol, with nickel identified a carcinogen present at higher concentrations in e-cigarette vapor than in combustible tobacco smoke (14, 17). Furthermore, e-cigarette use has also been linked to severe pulmonary injury and chronic respiratory conditions, including persistent cough, bronchitis, and dyspnea (18, 19). Surveys across multiple countries reveal significant knowledge gaps among young students: in the United States, only 20.1% of young students demonstrated adequate knowledge of e-cigarettes (20); in China, 45.0% of middle school students were aware of e-cigarettes (21). In other regions, data remain incomplete. Assessing the knowledge, attitudes, and practices (KAP) related to e-cigarettes among young students is therefore essential for developing targeted prevention strategies and evidence-based health policies.

Based on a systematic review of measurement tools in the field of e-cigarette research, the surveys primarily focus on assessing users’ perceptions, expectations, and beliefs regarding e-cigarettes (22, 23). This is followed by investigations into the motivations of non-users to try e-cigarettes or their future use intentions (24, 25); another significant area of research involves the assessment of e-cigarette dependence, including the Penn State Electronic Cigarette Dependence Index (PSECDI) (26). Most scales demonstrate acceptable internal consistency; however, validity testing is often limited, with most instruments evaluated through either exploratory factor analysis (EFA) or confirmatory factor analysis (CFA), rather than both (22, 27). Additionally, these tools often fail to account for youth-specific cultural and psychological contexts (22, 28), limiting comparability across studies.

To address these limitations and improve understanding of young student vaping behaviors, researchers have applied the KAP model to investigate e-cigarette use. As a well-established framework in health education and promotion, the KAP model describes the sequential process from knowledge acquisition to attitude development and ultimately behavioral adoption (29), providing a structured approach to identify cognitive determinants and guide behavioral interventions.

In psychometric research, Classical Test Theory (CTT) and Structural Equation Modeling (SEM) offer complementary methodological strengths. CTT focuses on item-level statistical indicators, such as difficulty, discrimination, and internal consistency, making it suitable for preliminary item screening and reliability testing due to its simplicity and interpretability (30). SEM, by contrast, provides a rigorous means of evaluating latent constructs through Confirmatory Factor Analysis (CFA), allowing assessment of convergent and discriminant validity and overall model fit (31).

Together, these theoretical and methodological foundations highlight the need for a standardized, psychometrically sound instrument to assess e-cigarette-related KAP among youth. Therefore, the present study aims to develop and validate an e-cigarette KAP questionnaire for young students using an SEM framework. By developing a theoretically grounded and empirically validated instrument, this study aims to establish a reliable measurement foundation for future research and policy-oriented public health interventions.

## 2 Methods

### 2.1 Measurements

The *E-cigarette KAP Questionnaire* for young students was developed following established psychometric principles for instrument construction. Based on the KAP theoretical framework, an initial pool of items was generated through a systematic literature review and expert brainstorming sessions. Six experts specializing in public health, health promotion, sociology, and cross-cultural psychology participated in a two-round Delphi consultation. All experts held at least an associate professor rank and had more than 5 years of professional experience in their respective domains.

The item-level content validity index (I-CVI) ranged from 0.86 to 1.00, and the scale-level content validity index (S-CVI) reached 0.94, well above the recommended threshold of 0.90, indicating excellent content relevance and semantic clarity.

After developing the Chinese version, professional linguists translated the questionnaire into English. Subsequently, Indonesian linguistic experts produced a culturally adapted Bahasa Indonesia version.

To ensure cross-cultural equivalence and linguistic clarity, a pilot test was conducted in April 2023 with 20 young students (10 from China and 10 from Indonesia). Cognitive interviews assessed item clarity, cultural appropriateness, and logical coherence. Based on participant feedback, minor modifications were made to ambiguous or culturally sensitive expressions.

A preliminary reliability assessment of the knowledge domain showed a Cronbach’s *α* of 0.99 and an inter-item Kappa coefficient of 0.69, demonstrating strong internal consistency and initial measurement stability, providing a foundation for large-scale validation.

### 2.2 Study design

A cross-sectional online survey was conducted between May and June 2023. Sampling sites were selected based on comparable smoking prevalence and urban demographics. Kunming, China (adult smoking rate: 33.7%) (32), and Jakarta, Indonesia (adult smoking rate: 32.2%) (33), were chosen as representative regions.

Sample sizes were calculated using standard formulas for cross-sectional surveys, yielding 567 and 553 participants from Kunming and Jakarta, respectively.


$$N=\frac{\mu_{\alpha }^{2}\times p(1-p)}{\delta^{2}}\times \text{deff}$$

A multistage stratified cluster sampling approach was employed. In each city, three universities were selected (one medical and two non-medical institutions). Within each institution, classes were randomly selected by grade level, and all students in the selected classes were invited to participate. Eligible participants were undergraduate students aged 18–25 years, currently enrolled at their universities, and willing to provide informed consent.

Ethical approval was obtained from the Ethics Committee of Kunming Medical University (Approval No. KMMU2022MEC027), and the study adhered to the principles of the Declaration of Helsinki (2013 revision).

### 2.3 Quality control

Data collection was conducted through an encrypted online platform. All questionnaire items were set as mandatory-response fields to minimize missing data. In pilot testing, the average completion time in pilot testing was 10 min (range: 8–12 min); responses completed in less than 3 min or more than 30 min were excluded as invalid. Each class participated under supervision coordinated by class advisors, following a standardized protocol. Instructions were read aloud to ensure procedural consistency, respondent independence, and a uniform testing environment. Data integrity was verified through double data entry and cross-checking by two independent researchers.

### 2.4 Item analysis

Item selection was guided by both CTT and SEM. Reliability testing, exploratory factor analysis (EFA), CFA, and discrimination analysis was combined to identify and remove items with weak psychometric performance or ambiguous factor loadings. The final instrument was refined to ensure clear theoretical dimensionality and measurement precision.

#### 2.4.1 Reliability analysis

Reliability was assessed using Cronbach’s *α*, with values ≥ 0.75 considered satisfactory and values ≥ 0.60 deemed acceptable for exploratory research purposes (34).

#### 2.4.2 Validity analysis

EFA was performed to evaluate structural validity and further refine the questionnaire. Principal component analysis with varimax rotation was conducted. Items were retained if they met all of the following criteria: (1) eigenvalue > 1; (2) scree plot inflection point; (3) factor loading > 0.4; (4) communality > 0.3; (5) at least three items per factor with strong internal consistency (35).

#### 2.4.3 Correlation coefficient method

The Pearson correlation coefficients between each item and its domain score were calculated. Items with item-total correlations below 0.4 were removed to ensure that each item measured the same construct as the overall scale (36).

#### 2.4.4 Critical ratio method

Participants were divided into high- and low-score groups based on the upper and lower 27% of total scores. Independent-sample *t*-tests were used to compare group mean differences for each item. Items with *t ≥ 3* or *p < 0.05* were considered discriminative and retained.

#### 2.4.5 Factor analysis

Items were subjected to principal component analysis with orthogonal varimax rotation. Items with factor loadings ≥ 0.4 were retained as indicators of satisfactory representativeness (37).

#### 2.4.6 Structural equation modeling (SEM)

CFA was conducted using the least squares estimation method to evaluate the factor structure. Model fit was assessed using the following indices: Bayesian Information Criterion (BIC), Comparative Fit Index (CFI), Tucker–Lewis Index (TLI), Standardized Root Mean Square Residual (SRMR), and Root Mean Square Error of Approximation (RMSEA) and its 90% confidence interval (38, 39). Criteria for acceptable model fit were: CFI and TLI > 0.90, RMSEA < 0.08 (excellent < 0.05), and SRMR < 0.08. Lower BIC values indicated better model parsimony.

##### 2.4.6.1 Model modification and theoretical constraints

Parameter modification was restricted to the release of error covariances between selected item pairs within the same latent construct, based on modification indices and theoretical justification. These item pairs shared similar wording or closely related behavioral or cognitive content, suggesting correlated measurement error attributable to method effects rather than construct overlap. These pairings were selected based on high modification indices and a substantive rationale (e.g., shared wording or thematic content indicating possible common method variance). Critically, no cross-loadings or between-construct error covariances were added, ensuring the original three-factor KAP framework remained intact. Refinement was conducted conservatively, prioritizing theoretical coherence and parsimony over optimal fit statistics.

### 2.5 Statistical analysis

Data were entered into Microsoft Excel 2019 and analyzed using SPSS 27.0 and AMOS 29.0 software. Categorical variables were reported as frequencies and percentages. Continuous variables conforming to a normal distribution were described as *mean ± standard deviation (SD)*; non-normally distributed variables were reported as *median (P25, P75)*. A two-tailed *p*-value < 0.05 was considered statistically significant.

## 3 Results

### 3.1 Sample characteristics

A total of 1,400 questionnaires were distributed, and 1,333 valid responses were obtained after excluding incomplete and invalid submissions (631 from Kunming and 702 from Jakarta), yielding an effective response rate of 95.2%. Among the respondents, 594 (44.6%) were male and 739 (55.4%) were female; 47.3% were Chinese and 52.7% Indonesian. By grade level, 549 (41.8%) were freshmen, 582 (44.3%) sophomores, and 182 (13.7%) juniors or above. Regarding academic major, 412 (30.9%) were medical students, and 921 (69.1%) were non-medical.

With respect to religion, 731 (54.8%) reported religious affiliation and 602 (45.2%) did not. Urban residents accounted for 62.6% of the sample, while 37.4% were from rural areas. Monthly living expenses were <1,000 yuan for 31.4%, 1,000–1,999 yuan for 41.4%, and ≥2,000 yuan for 27.2%. Additionally, 8.7% had family members who used e-cigarettes, 29.3% had close friends who used them, and 74.3% had been exposed to secondhand smoke within the past week.

Based on literature review, expert consultation, and pilot testing, the initial scale consisted of 32 items (9 knowledge, 15 attitude, 8 behavior items), with the total score of 40 points (knowledge: 9; attitude: 15; behavior: 16). The mean total score was 18.76 ± 2.23, with subscale means of 5.74 ± 1.38 (knowledge), 12.30 ± 3.24 (attitude), and 6.89 ± 2.22 (behavior).

### 3.2 Item screening

#### 3.2.1 Critical ratio method

Analysis revealed that five behavior items (P2, P3, P4, P6, and P8) had critical ratios below 3.0, with non-significant differences (*p* > 0.05) between high- and low-scoring groups.

#### 3.2.2 Correlation coefficients

Item–total correlations ranged from 0.027 to 0.562. In the knowledge domain, items K2 and K3 showed correlations < 0.4 with their respective dimensions, while all other items exceeded 0.4 (*p* < 0.05). In the attitude domain, item A11 had a correlation < 0.4. In the behavior domain, P4, P6, P7, and P8 fell below this threshold.

#### 3.2.3 Factor analysis

Principal component analysis indicated that all item loadings exceeded 0.4, ranging from 0.416 to 0.789, demonstrating satisfactory factor structure.

#### 3.2.4 Cronbach’s *α* coefficients

The overall Cronbach’s *α* was 0.397; for the subscales, *α* was 0.394 for knowledge, 0.856 for attitude, and 0.160 for behavior. Sequential deletion analysis within the knowledge domain showed Cronbach’s *α* coefficients ranged from 0.237 to 0.500, with internal consistency improving when items K2 and K3 were removed. For the attitude domain, removal of individual items resulted in α coefficients ranging from 0.839 to 0.855, indicating minimal impact on reliability. Within the behavior domain, deletion of individual items produced α coefficients ranging from 0.014 to 0.442; notably, reliability improved when items P4 and P8 were removed.

Given these findings, items K2, K3, P4, P6, and P8 were deleted to optimize internal consistency. The revised questionnaire retained 27 items (knowledge = 7, attitude = 15, behavior = 5). The revised structure is shown in Table 1.

**Table 1:** Results of item selection for the questionnaire.

Item	Critical ratio (CR)	*p-*value	corrected item-total correlation	*p*-value of the correlation coefficient	Correlation with the total questionnaire score	*p*-value of the correlation coefficient	factor loading	Cronbach’s alpha if item deleted	Item evaluation
K1	11.961	<0.05	0.533	<0.05	0.359	<0.05	0.668	0.300	Retain
K2	−6.158	<0.05	0.091	<0.05	−0.158	>0.05	0.669	0.500	Delete
K3	−5.927	<0.05	0.096	<0.05	−0.145	>0.05	0.601	0.499	Delete
K4	9.224	<0.05	0.645	<0.05	0.061	>0.05	0.737	0.237	Retain
K5	10.346	<0.05	0.568	<0.05	0.097	>0.05	0.757	0.300	Retain
K6	10.235	<0.05	0.469	<0.05	0.163	>0.05	0.579	0.372	Retain
K7	13.619	<0.05	0.497	<0.05	0.305	<0.05	0.537	0.324	Retain
K8	8.425	<0.05	0.438	<0.05	0.341	<0.05	0.775	0.340	Retain
K9	7.647	<0.05	0.435	<0.05	0.322	<0.05	0.640	0.337	Retain
A1	9.346	<0.05	0.656	<0.05	0.471	<0.05	0.656	0.844	Retain
A2	11.285	<0.05	0.680	<0.05	0.527	<0.05	0.789	0.842	Retain
A3	14.311	<0.05	0.709	<0.05	0.562	<0.05	0.700	0.839	Retain
A4	11.538	<0.05	0.663	<0.05	0.471	<0.05	0.729	0.842	Retain
A5	15.307	<0.05	0.652	<0.05	0.480	<0.05	0.665	0.843	Retain
A6	15.713	<0.05	0.545	<0.05	0.100	>0.05	0.713	0.853	Retain
A7	13.864	<0.05	0.493	<0.05	0.092	>0.05	0.680	0.852	Retain
A8	12.589	<0.05	0.547	<0.05	0.242	<0.05	0.759	0.849	Retain
A9	13.469	<0.05	0.648	<0.05	0.558	<0.05	0.630	0.843	Retain
A10	16.009	<0.05	0.580	<0.05	0.337	<0.05	0.579	0.848	Retain
A11	5.498	<0.05	0.379	<0.05	0.250	<0.05	0.755	0.855	Retain
A12	13.375	<0.05	0.609	<0.05	0.523	<0.05	0.619	0.846	Retain
A13	24.311	<0.05	0.633	<0.05	0.190	<0.05	0.416	0.846	Retain
A14	10.7	<0.05	0.464	<0.05	−0.029	>0.05	0.602	0.854	Retain
A15	8.953	<0.05	0.423	<0.05	0.260	<0.05	0.719	0.855	Retain
P1	2.315	<0.05	0.557	<0.05	0.129	>0.05	0.559	−0.014	Retain
P2	−0.287	>0.05	0.478	<0.05	0.064	>0.05	0.611	0.096	Retain
P3	1.610	>0.05	0.611	<0.05	0.180	<0.05	0.475	−0.075	Retain
P4	0.009	>0.05	0.320	<0.05	0.027	>0.05	0.474	0.214	Delete
P5	3.591	<0.05	0.461	<0.05	0.315	<0.05	0.710	0.055	Retain
P6	1.398	>0.05	0.367	<0.05	0.214	<0.05	0.799	0.142	Delete
P7	3.880	>0.05	0.346	<0.05	0.303	<0.05	0.664	0.141	Retain
P8	0.625	>0.05	0.096	>0.05	0.063	>0.05	0.446	0.442	Delete

### 3.3 Reliability analysis

Cronbach’s *α* for the total scale was 0.605, while the *α* values for the knowledge, attitude, and behavior subscales were 0.702, 0.856, and 0.775, respectively, indicating acceptable to good internal consistency. The total scale reliability (*α* = 0.605) falls within the range deemed acceptable for initial research applications of a multidimensional tool.

### 3.4 Confirmatory factor analysis (CFA)

The SEM framework was applied to evaluate the model’s fit and theoretical consistency. Three candidate models (M1–M3) were compared: Model M1, a basic three-factor model without cross-loading; Model M2, a three-factor correlated model with adjusted measurement errors; Model M3, a modified SEM model after iterative parameter optimization.

The final model (M3) exhibited the best fit: *χ^2^/df* = 1.350, *RMSEA* = 0.050 (*90% CI:* 0.022–0.072), *SRMR* = 0.0643, *CFI* = 0.968, *TLI* = 0.960 (see Table 2).

**Table 2:** Fit indices for the structural model of the e-cigarette KAP questionnaire.

Model	*χ^2^*	*df*	*χ^2^/df*	RMSEA (*90%CI*)	SRMR	NFI	TLI	CFI	AIC	BIC
M1	763.275	249	3.065	0.122(0.112–0.132)	0.1359	0.540	0.588	0.629	913.275	946.170
M2	161.050	87	1.851	0.078(0.059–0.097)	0.0697	0.844	0.904	0.920	257.050	269.538
M3	114.744	85	1.350	0.050(0.022–0.072)	0.0643	0.889	0.960	0.968	214.744	227.752

**Figure 1:**
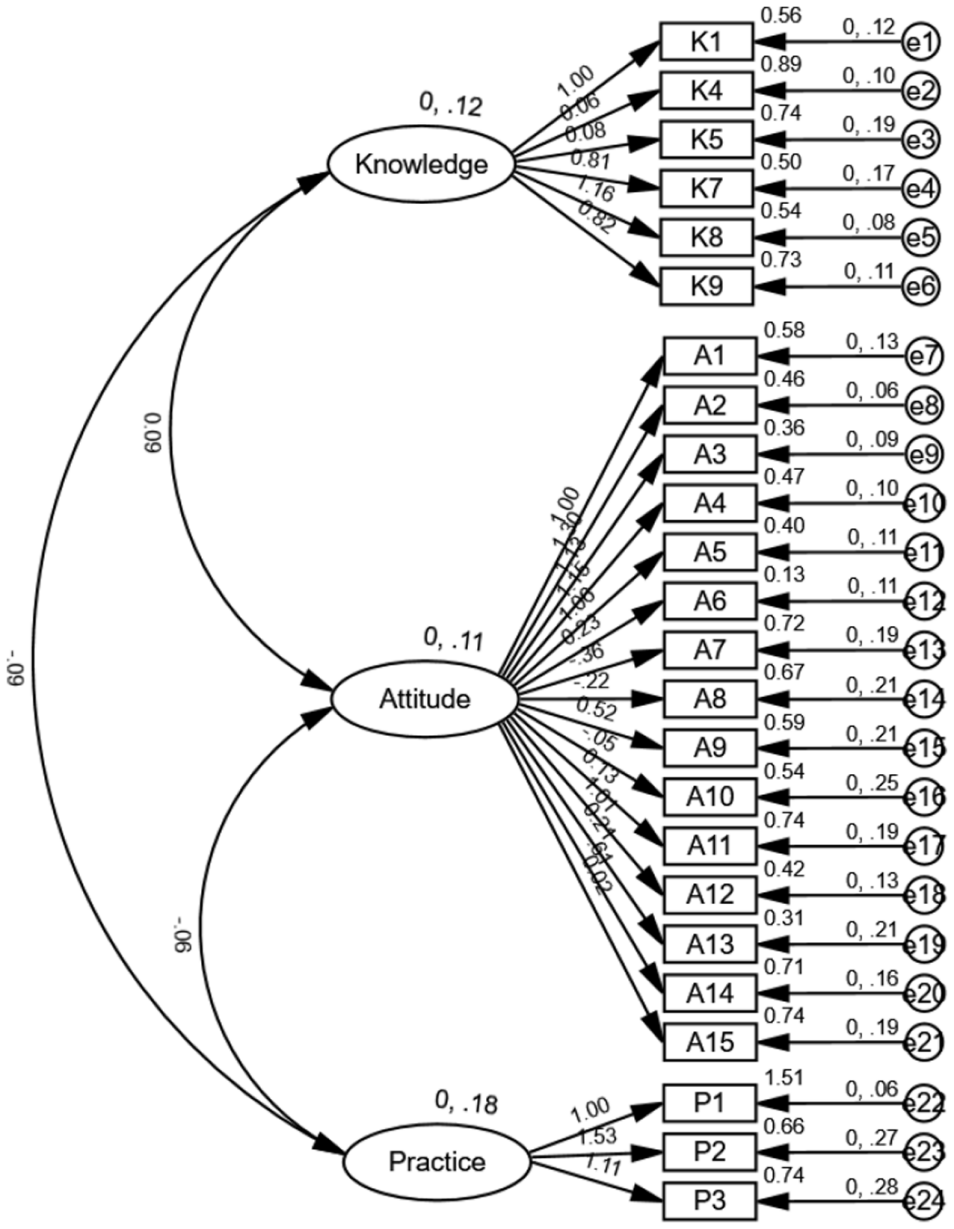
Initial model of the confirmatory factor analysis for the e-cigarette KAP scale.

**Figure 2:**
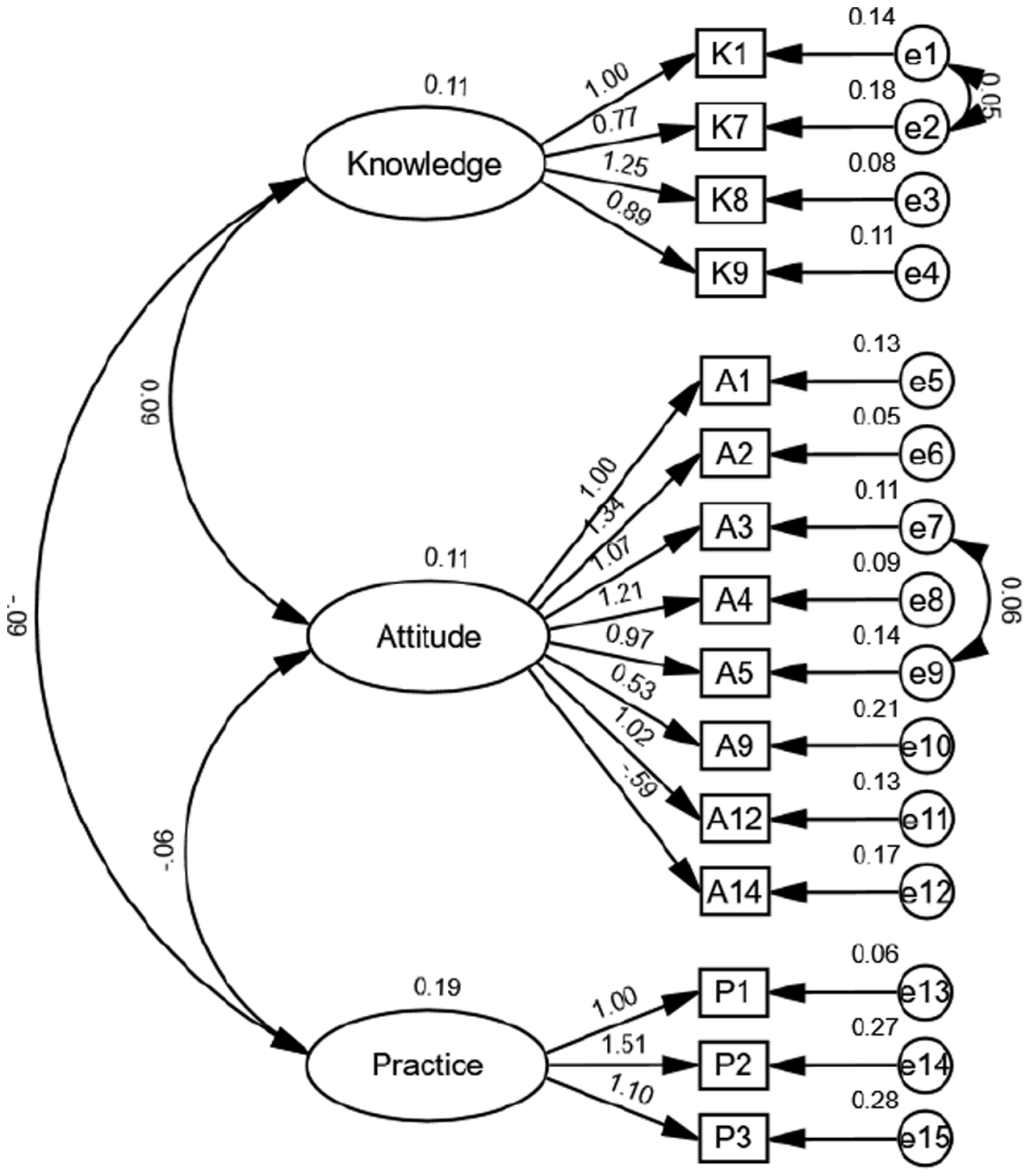
Final model of the confirmatory factor analysis for the e-cigarette KAP scale.

All fit indices met or exceeded the recommended thresholds (*CFI*, *TLI* > 0.90; *RMSEA* < 0.08), indicating excellent model fit and parsimony. Standardized factor loadings ranged from 0.52 to 0.84, confirming strong relationships between latent variables and observed indicators. The initial and final model structure is presented in Figures 1, 2, respectively.

### 3.5 Model fit evaluation

Comprehensive evaluation of the model’s internal structure demonstrated that the knowledge-attitude correlation was *r* = 0.682 (*p* < 0.001); the attitude-practice correlation was *r* = 0.715 (*p* < 0.001); the knowledge-practice correlation was *r* = 0.639 (*p* < 0.001). These findings indicate moderate associations among the three dimensions, aligning with the theoretical logic of the KAP model, namely, that knowledge influences attitudes, which in turn drive behavioral practice.

### 3.6 Reliability and internal consistency

The overall Cronbach’s *α* coefficient of the questionnaire was 0.643, with the subscale coefficients as follows: knowledge = 0.819, attitude = 0.847, and practice = 0.775. All subscale coefficients exceeded the recommended threshold of 0.70. Although the overall Cronbach’s *α* of the questionnaire was slightly below 0.7, it remains acceptable for early-stage psychometric instrument research. When combining the knowledge and attitude subscales, the Cronbach’s *α* coefficient was 0.889. In contrast, when knowledge, attitude, and practice were analyzed together, the overall Cronbach’s α of the questionnaire was 0.643. Split-half reliability further confirmed the internal consistency, indicating that each subscale reliably measures its intended construct.

### 3.7 Validity assessment

Convergent validity was verified through factor loadings and composite reliability (CR). All standardized loadings exceeded 0.50, and Average Variance Extracted (AVE) values for each factor were above 0.50, satisfying *Fornell–Larcker* criteria for convergent validity. Discriminant validity analysis showed that the square root of AVE for each dimension was greater than its inter-factor correlations, indicating good construct separation and theoretical distinctness.

### 3.8 Summary of the final scale

Following reliability and validity optimization, the final E-cigarette Knowledge, Attitude, and Practice Questionnaire consisted of 15 items distributed across three dimensions: (1) Knowledge—4 items, (2) Attitude—8 items, (3) Practice—3 items.

The model structure was both statistically stable and theoretically consistent with the KAP framework, providing a scientifically sound foundation for subsequent cross-cultural and behavioral studies.

## 4 Discussion

### 4.1 Principal findings

This study constructed and psychometrically evaluated an e-cigarette KAP Questionnaire for young students in China and Indonesia using an SEM framework. The final 15-item scale demonstrates acceptable reliability and construct validity. The three-factor structure (knowledge, attitude, and practice) was empirically supported and the final model (M3) achieved excellent fit indices (*χ*^2^/df = 1.350, RMSEA = 0.050, CFI = 0.968, TLI = 0.960). Importantly, model refinement was limited to theoretically justified within-factor error covariances, with no cross-loadings or cross-factor error correlations introduced, thereby preserving the conceptual integrity of the KAP framework. These findings validate the theoretical proposition of the KAP model that cognitive understanding influences behavioral intentions and practices through attitudinal pathways (29). Moreover, the positive correlations observed among the three domains (*r* = 0.639–0.715) indicate that higher e-cigarette-related knowledge is associated with more rational attitudes and healthier behavioral tendencies, consistent with previous studies on smoking cognition and prevention among young students (40, 41).

The final Practice subscale was distilled into three core items through Classical Test Theory and Structural Equation Modeling: P1. Have you ever used e-cigarettes? P2. In the past 7 days, on how many days did you use e-cigarettes? P3. On average, how many times per day do you use e-cigarettes? These items capture the fundamental dimensions of use behavior: lifetime adoption (P1), recent engagement (P2), and use intensity (P3). This minimal set efficiently classifies respondents into public health-relevant categories, such as never-users, experimenters, occasional users, and regular users. However, this set cannot fully reflect all aspects of use behavior. Therefore, we retained item P8 (What is your primary reason for using e-cigarettes?) as an open-ended inquiry into motivations, which aids in the interpretability of the quantitative findings across different cultural contexts.

Psychometric analysis indicated that the combined knowledge and attitude subscales yielded a Cronbach’s *α* of 0.889. However, when the practice subscale was included, the overall reliability of the scale dropped to a moderate level (*α* = 0.643). The measurement of sensitive behaviors like e-cigarette use among youth necessitates a critical appraisal of potential biases and the sociocultural contexts that shape both behavior and reporting. First, social desirability bias likely influenced responses, particularly on the ‘Practice’ items. Despite our use of anonymous surveys to minimize this bias, participants may still have underreported use to conform to perceived social expectations. Second, cultural differences between China and Indonesia may underlie the divergence in e-cigarette use patterns among young students. Social desirability and policy environments may exert differential influences on self-reporting. In China, where tobacco control policies and school anti-smoking norms are widely implemented, respondents may underreport or deny e-cigarette use to align with societal expectations of being a “good student” or a “law-abiding citizen” (42, 43). In contrast, within the socioeconomic and cultural context of Indonesia, the tobacco industry wields substantial economic influence, adult smoking prevalence is high, and e-cigarettes lack clear regulation. Tobacco products are widely available through various retail channels, such as small stalls and street vendors, making them easily accessible to young students (44, 45). Therefore, future applications of this scale must account for the profound influence of local policy environments and socio-cultural norms on behavioral reporting. This insight is a principal finding of our validation study, highlighting that the instrument’s value lies not only in measuring constructs but also in revealing the contextual boundaries of those measurements.

### 4.2 Comparison with previous studies

The findings align with international evidence suggesting that knowledge and attitude are key determinants of youth e-cigarette behaviors (46). Prior research has shown that young students with insufficient awareness of nicotine dependence or e-cigarette toxicity are more likely to engage in experimental or routine vaping (47) whereas high knowledge and unfavorable attitudes toward e-cigarettes as protective factors against initiation (47, 48).

This study extends these findings by confirming that these relationships remain stable across culturally distinct populations, namely China and Indonesia. Notably, while earlier instruments have primarily emphasized behavioral dependence (e.g., Penn State Electronic Cigarette Dependence Index) (26, 49), our scale integrates cognitive, affective, and behavioral dimensions, enabling a more comprehensive assessment of youth perceptions. Furthermore, the SEM-based validation approach enhances methodological rigor beyond traditional CTT tools, strengthening interpretability and facilitating cross-cultural comparison.

### 4.3 Methodological strengths and public health implications

A key strength of this study is the methodological integration of CTT and SEM. The complementary nature of the two psychometric paradigms ensured rigorous construct validation and optimized item selection, an approach consistent with contemporary measurement practices (30, 31).

Theoretically, the present study the results provide empirical support for the sequential mechanism posited by the KAP model, knowledge shapes attitudes, which in turn influence behavioral practices, clarifying how health education affects preventive behavior. From a public health perspective, this questionnaire provides a reliable and evidence-based tool for assessing youth cognition and behavior toward e-cigarettes. It can be used to: (1) evaluate the effectiveness of health education interventions, (2) identify high-risk groups with low awareness or misperceptions, and (3) inform school- and community-based tobacco control strategies.

### 4.4 Limitations and future directions

Several limitations should be acknowledged. First, the participants were young students from two cities, which limits generalizability to younger young students or non-student groups. Future research should expand sampling to secondary school students and working young adults to capture broader behavioral diversity. Second, although SEM provides comprehensive structural verification, temporal stability (test–retest reliability) and predictive validity were not assessed; future longitudinal research is warranted. Third, this study did not formally assess cross-cultural equivalence. Although the instrument showed acceptable psychometric properties, measurement invariance across cultures has not been established. Cross-cultural adaptation and comparability will be addressed in a separate forthcoming study. Finally, as with most self-reported surveys, responses may be influenced by social desirability bias. Future work ought to implement this questionnaire in intervention and surveillance contexts to monitor cognitive and behavioral trends over a period of time. Additional validation of the instrument should be conducted across a greater variety of countries with larger sample sizes, in order to enhance its cross-cultural applicability and underpin evidence-based global efforts in e-cigarette control.

## 5 Conclusion

This study developed and validated an E-cigarette KAP Questionnaire among young students in China and Indonesia using both CTT and SEM approaches. The final 15-item instrument demonstrated acceptable reliability and validity within the study sample. Its three-factor structure (knowledge, attitude, and practice) was empirically substantiated, and the SEM model exhibited excellent fit indices. The findings confirm the theoretical foundation of the KAP framework: knowledge acquisition enhances attitudes, which subsequently influence behavior. By integrating psychometric rigor with public health application, this study provides a practical measurement tool for assessing e-cigarette-related cognition and behaviors among young students. The validated questionnaire can serve as an effective instrument for monitoring awareness, guiding educational interventions, and informing evidence-based tobacco-control policies in diverse cultural contexts. Future research should test the scale in longitudinal and interventional contexts to better understand cognitive and behavioral dynamics related to e-cigarette use.

## Data Availability

The datasets presented in this article are not readily available because the data supporting this study are not publicly available due to participant privacy concerns. However, data can be obtained from the corresponding author upon reasonable request. Requests to access the datasets should be directed to RD dengruirita@126.com.

## Appendix

### Appendix A1: SEM model modification strategy

To ensure theoretical rigor and avoid data-driven overfitting, the following predefined principles guided SEM model modification:

1. Structural invariance
  The latent factor structure (knowledge, attitude, and practice) was fixed across all models (M1–M3). No item was reassigned to a different latent construct.
2. No cross-loadings
  Cross-loadings were not permitted at any stage of model refinement.
3. Restricted error covariance specification
  Error covariances were considered only between item pairs within the same latent construct, and only when supported by both modification indices and clear conceptual similarity (e.g., overlapping wording or shared behavioral context).
4. No cross-factor error correlations
  Error covariances between items from different latent constructs were explicitly prohibited to preserve construct distinctiveness.
5. Iterative but conservative refinement
  Model modifications were introduced sequentially, and each step was evaluated based on theoretical plausibility, improvement in global fit indices, and model parsimony.

This constrained approach ensured that Model M3 represents a theory-driven refinement of the initial measurement model rather than an exploratory or data-driven optimization.
